# A review of *Listeria monocytogenes* from meat and meat products: Epidemiology, virulence factors, antimicrobial resistance and diagnosis

**DOI:** 10.4102/ojvr.v87i1.1869

**Published:** 2020-10-09

**Authors:** Itumeleng Matle, Khanyisile R. Mbatha, Evelyn Madoroba

**Affiliations:** 1 Bacteriology Division, Agricultural Research Council – Onderstepoort Veterinary Research, Onderstepoort, Pretoria, South Africa; 2 Department of Agriculture and Animal Health, University of South Africa, Science Campus, Florida, South Africa; 3 Department of Biochemistry and Microbiology, University of Zululand, KwaDlangezwa, South Africa

**Keywords:** Listeria monocytogenes, meat and meat products, epidemiology, virulence factors, diagnosis, antimicrobial resistance

## Abstract

*Listeria monocytogenes* is a zoonotic food-borne pathogen that is associated with serious public health and economic implications. In animals, *L. monocytogenes* can be associated with clinical listeriosis, which is characterised by symptoms such as abortion, encephalitis and septicaemia. In human beings, listeriosis symptoms include encephalitis, septicaemia and meningitis. In addition, listeriosis may cause gastroenteric symptoms in human beings and still births or spontaneous abortions in pregnant women. In the last few years, a number of reported outbreaks and sporadic cases associated with consumption of contaminated meat and meat products with *L. monocytogenes* have increased in developing countries. A variety of virulence factors play a role in the pathogenicity of *L. monocytogenes*. This zoonotic pathogen can be diagnosed using both classical microbiological techniques and molecular-based methods. There is limited information about *L. monocytogenes* recovered from meat and meat products in African countries. This review strives to: (1) provide information on prevalence and control measures of *L. monocytogenes* along the meat value chain, (2) describe the epidemiology of *L. monocytogenes* (3) provide an overview of different methods for detection and typing of *L. monocytogenes* for epidemiological, regulatory and trading purposes and (4) discuss the pathogenicity, virulence traits and antimicrobial resistance profiles of *L. monocytogenes*.

## *Listeria* species

*Listeria* species (spp.) are short Gram-positive rods (Nyarko & Donnelly 2015) that belong to the phylum *Firmicute*s, class *Bacilli*, order *Bacillales* (Jadhav 2015). The *Listeria* spp. are facultatively anaerobic, non-spore forming, about 0.5 *µ*m in width and 1 *µ*m – 1.5 *µ*m in length (Wieczorek, Dmowska & Osek 2012) and belong taxonomically to the *Clostridium-Bacillus-Lactobacillus* sub-branch with *Brochothrix thermosphacta* (Schmid et al. 2014). *Listeria* spp. are generally motile because of peritrichous flagella at temperature range of 24 °C – 28 °C but non-motile above 30 °C (Indrawattana et al. 2011). These species are also catalase positive; however, exceptions have been reported (Cepeda et al. 2006). *Listeria* spp. are oxidase, urea and indole negative and hydrolyse aesculin (De Vasconcelos Byrne et al. 2016). *Listeria* spp. also have the ability to tolerate salt conditions (NaCl) up to 20% (weight/volume [w/v]) and grows in a pH range of 4.4–9.6 (Holch et al. 2013). The growth temperature for these species ranges from –0.4 °C to 45 °C, with an optimum growth temperature of 37 °C and can survive at relatively low water activities (*aw* < 0.90) (Liu 2006). These growth conditions contribute to their versatility to grow and survive under extreme environmental conditions posed at food-processing facilities and become a serious problem for food industry (Ducey et al. 2006).

The genus *Listeria* consists of 20 low guanine (G) + cytosine (C) (38%) content species, which includes *L. monocytogenes, L. marthii, L. innocua, L. welshimeri, L. seeligeri, L. costaricensis, L. ivanovii, L. grayi, L. rocourtiae, L. fleischmannii, L. newyorkensis, L. weihenstephanensis, L. floridensis, L. aquatica, L. thailandensis, L. cornellensis, L. riparia, L. booriae, L. Goaensis* and *L. Grandensis* (Den Bakker et al. 2010). This classification of *Listeria* spp. is based on different analysis assays including 16S ribosomal ribonucleic acid (rRNA), deoxyribonucleic acid (DNA) sequencing information and multilocus enzyme analysis. It has also been proved useful for surveillance and epidemiological purposes in outbreaks linked to food-borne listeriosis (Chasseignaux et al. 2001). Out of the species of *Listeria* identified thus far, only *L. monocytogenes* can cause infection in both humans and animals, whereas *L. ivanovii* is pathogenic to animals, predominantly in ruminants and has rarely been implicated with human infections (Gouin et al. 1994; Orsi, Bakker and Wiedmann 2011; Zhang, Jayarao & Knabel 2004). However, *L. Seeligeri* and *L. welshimeri* have been reported as agents of sporadic cases of human listeriosis (Gouin et al. 1994).

*Listeria* spp. are phenotypically very similar but can be differentiated by biochemical tests including haemolysis test, acid production from D-xylose, L-rhamnose, mannitol, motility and alpha methy-D mannoside (Orsi et al. 2011). The Christie–Atkins–Munch-Peterson test can also be useful for differentiation of *Listeria* spp. This test works on the principle to test for haemolysis enhancement in the presence of *Staphylococcus aureus* (FDA et al. 2008).

*Listeria* spp. are mostly environmental contaminants with primary habitation of soil. However, these species have also been found in water, sewage and decaying vegetation (Willaarts, Pardo & De la Mora 2013). *Listeria* spp. have been found in a variety of animals including ruminants, birds, marine life, insects, ticks and crustaceans (Van Vuuren 2001). These species have been isolated from 1% to 7% of intestinal content of healthy animals (Borucki & Call 2003); thus animals can serve as carriers. Human beings are also carriers of *Listeria* spp. as these were isolated from 5% to 10% of stools of healthy human adults and 1.3% of the younger people (Churchill, Lee & Hall 2006). As a result of their ubiquitous and resilient character, *Listeria* spp. have the capacity to enter the food supply chain and contaminate a wide variety of food products (Leong, Alvarez-Ordóñez & Jordan 2014).

### Listeria monocytogenes

*Listeria monocytogenes* was first described by Hülphers in 1910 from the necrotic liver of a rabbit in Sweden and named *Bacillus hepatis* (Carvalho, Sousa & Cabanes 2014; Hülphers 1911). Murray isolated a similar bacterium in 1926 as a causative agent of an epizootic in rabbits and guinea pigs in research laboratories of Cambridge, United Kingdom, and named *Bacterium monocytogenes* (Lekkas 2016; Murray, Webb & Swann 1926; Rantsiou et al. 2008). A year later (1927), Pirie also isolated a bacterium corresponding to the description given by Hülphers and Murray from wild gerbils in South Africa (SA) (Jemal 2014; Mitchell, Pirie & Ingram 1927). The bacterium was named *Listerellahepatolytica* in honour of British surgeon, Lord Joseph Lister, the father of antisepsis (Gray & Killinger 1966). However, it was until 1940, that the present name, *L. monocytogenes* was recognised (Lamont & Sobel 2011).

*Listeria monocytogenes* was first isolated in humans by Nyfeldt in 1929, and in the same year Gill also described the illness in sheep called circling diseases caused by *L. monocytogenes* (Gill et al. 1937; Nyfeldt 1929). *Listeria monocytogenes* was then recognised as pathogen that caused sporadic human infections and was mainly associated with workers encountering diseased animals (Lamont & Sobel 2011). In the 1980s, after several outbreaks including Vacherin Mont d’Or in Switzerland in 1983–1987 and improperly pasteurised milk in the United States (US) in 1983, that was when interest for the pathogen amongst food manufacturers started to emerge (Klumpp & Loessner 2013; Lekkas 2016). Since then, *L. monocytogenes* outbreaks have been linked to consumption of contaminated foods, which include dairy products, meat products, seafood products and vegetables (Loman 2012; Ragon et al. 2008; Zuber et al. 2019).

*Listeria monocytogenes* is amongst the dangerous bacterial food-borne pathogens in the world, which cause severe human diseases (Maertens de Noordhout et al. 2014). This pathogen has been divided into 13 serotypes (½a, ½b, ½c, 3a, 3b, 3c, 4a, 4ab, 4b, 4c, 4d, 4e, 7) based on somatic and flagellar antigens (Dhama et al. 2015). These serotypes are further grouped into four genetic diversity lineages (I–IV). These lineages consist of specific serotypes; lineage I harbours serotypes ½b, 3b, 4b, 4d, 4e and 7. Serotypes ½b and 4b within lineage I encode listeriolysin S virulence factor, which is not present in other lineages (Orsi et al. 2011). Lineage II contains serotypes ½a, ½c, 3a and 3c and often harbours several plasmids that are resistant to heavy metals (Dhama et al. 2015). Serotypes 4b, ½a, 4a and 4c belong to lineage III and 4a, 4c and atypical 4b serotypes have been characterised as lineage IV isolates (Haase et al. 2014). Lineage III and IV serotypes are rarely isolated and have distinctive genetic and phenotypic characteristics but occur mostly in ruminants (Camargo, Woodward & Nero 2016).

## Human listeriosis

Listeriosis is a zoonotic disease that is mainly acquired through consumption of contaminated food by *L. monocytogenes* (Hilliard et al. 2018). Other possible routes of contamination for humans include direct contact with infected animals and environments (Vázquez-Boland et al. 2001). Uncommon occurrence of listeriosis has also been reported in human beings in the form of endocarditis, hepatitis, myocarditis, arteritis, pneumonia, sinusitis, conjunctivitis, ophthalmitis and joint infections (Amato et al. 2017). The incidences of listeriosis are very low in general population, but it remains a major and deadly food-borne disease with hospitalisation rate of over 95% (Scallan et al. 2011). This disease occurs in specific segments of the population, which are the elderly, pregnant women, unborn babies and immunocompromised people such as those suffering from acquired immune deficiency syndrome (AIDS) or cancer or those who have undergone organ transplants (Maertens de Noordhout et al. 2014).

Listeriosis is characterised by a wide spectrum of infections, which are categorised into two forms, namely severe invasive listeriosis and non-invasive febrile gastroenteritis (Buchanan et al. 2017). Invasive listeriosis mostly occurs in immunocompromised individuals and manifests itself as sepsis, meningitis, endocarditis, encephalitis, meningoencephalitis, septicaemia and brain infection (Doganay 2003). The brain infection because of listeriosis in immunocompromised adults is responsible for 22% fatalities, whereas endocarditis occurs in 10% of adults (Mizuno et al. 2007). Pregnant woman have 17-fold increased risk of contracting invasive listeriosis, and this infection mostly occurs in the third trimester (Mateus et al. 2013). Listeriosis in pregnant women is generally associated with flu-like symptoms with or without gastrointestinal problem (Doganay 2003). However, the consequences of foetus or newborn infection are extremely severe, which includes abortion, premature birth, pneumonia and meningitis (Indrawattana et al. 2011). Invasive listeriosis is responsible for over 90% of hospitalisation and between 20% and 30% case fatality rate (Leong et al. 2014), making it one of the most serious food-borne diseases.

Non-invasive gastroenteritis can manifest in immunocompetent adults and usually causes atypical meningitis, septicaemia and febrile gastroenteritis characterised by fever and watery diarrhoea lasting for 2–3 days, which is often accompanied by headache and backache (Mateus et al. 2013). These symptoms are usually self-limiting and can be resolved within a short period without seeking any medical attention, subsequently leading to undiagnosed and under reporting of cases (Matle 2016). In addition, the health workers are unlikely to report condition associated with non-invasive listeriosis because of their less severe manifestation when individuals seek medical attention. This poses a challenge to the health care system to maintain the knowledge and diagnose listeriosis cases especially in the developing world (Maertens de Noordhout et al. 2014).

The expression of both forms of listeriosis depends on the age of the individual, immune status of the individual, infectious dose and mode of infection, virulence of strain ingested and physiological stage (Poimenidou et al. 2018). The clinical signs of this disease often appear after a long incubation time (1 day – 70 days), which make epidemiological source tracing very difficult (Buchanan et al. 2017). Goulet et al. (2008) reported that incubation periods are largely influenced by clinical forms of the disease, with the longest incubation periods observed in pregnancy cases (median 27.5 days), followed by the central nervous system infection (median 9 days), sepsis (median 2 days) and febrile gastrointestinal disease (median 24 h). The estimated infective dose for listeriosis to occur in susceptible population is 0.1 to 10 million colony-forming units (cfu), whereas in healthy individual it is 10 to 100 million cfu (Angelo et al. 2017).

A study performed by Nappi et al. (2005) indicated that molecular characterisation of *L. monocytogenes* using serotyping allowed for the identification of the serotypes ½a, ½b and 4b as the predominate causative agents of listeriosis in humans. Furthermore, several studies showed that most human listeriosis outbreaks have been associated with *L. monocytogenes* serotype 4b, suggesting specific virulence properties in this serotype (De Cesare et al. 2001; Salcedo et al. 2003; Vasconcelos et al. 2008). Serotyping of *L. monocytogenes* isolates have also shown that serotypes belonging to antigenic group ½ (½a, ½b, ½c) have been over-represented in food isolates with sharp increase in clinical human isolates (Vázquez-Boland et al. 2001). No association have been established between certain forms of listeriosis and specific serotypes, but studies suggest a link between perinatal listeriosis and serotypes ½a and 4b (Soni et al. 2014). In Europe and North America, most human listeriosis cases over the past 20 years (2000–2010) involved serotype 4b and it was shown to be over-represented in perinatal listeriosis (Lacroix et al. 2014).

The four defined lineages (I, II, III and IV) of the *L. monocytogenes*, lineage I and II isolates have been associated with the majority of human listeriosis outbreaks and concurrent sporadic cases in the world (Schmitz-Esser et al. 2015). This suggests that serotypes harboured by these lineages have an increased virulence or a better adaptation to the human host (Corde et al. 2018). The distribution of these lineages varies by region with lineage II isolates mostly common amongst human listeriosis cases in Europe (Martín et al. 2014), compared with the US where lineage I strains seem to be predominant amongst human listeriosis cases (Roberts et al. 2018). The difference in geographical distribution of these lineages is linked to epidemic clones (ECs). Epidemic clones are closely related strains, associated with several geographically and temporally distinct listeriosis outbreaks (Chen & Knabel 2007). These ECs are also implicated in many human listeriosis outbreaks and contribute significantly to sporadic cases worldwide (Scortti et al. 2018). The ECI and ECII were each responsible for repeated human listeriosis in the US and Europe (Mammina et al. 2013). The ECIII caused a multistate outbreak associated with contaminated turkey (Chen & Knabel 2007).

Many food-borne listeriosis outbreaks have been linked to diverse food products, but different types of meat have been implicated in major human listeriosis outbreaks worldwide. Table 1 gives an overview of the listeriosis outbreaks associated with meat products during 1987–2018. The first laboratory-confirmed invasive case of listeriosis associated with meat products occurred in 1988 because of consumption of contaminated turkey franks (Schwartz et al. 2018). Since then, the vast majority of meat products were involved in listeriosis outbreaks or sporadic cases and included processed, vacuum-packaged meat products (Cases et al. 2016; Chen et al. 2017), pork tongue (Bozzuto, Ruggieri & Molinari 2010), sausages (Cases et al. 2016) and polony (Smith et al. 2019). The largest documented outbreaks of listeriosis in SA occurred between 2017 and 2018. This outbreak was associated with consumption of polony, which is a ready-to-eat (RTE) meat product and serotype 4b was the predominant isolate (Smith et al. 2019).

**TABLE 1:** Major food-borne listeriosis outbreaks because of meat products in the world.

Year	Country	No. of cases (death)	Meat type	Serotype
1987–1989	UK	366 (ND)	Paté	4b
1900	Australia	9 (6)	Processed meats, paté	ND
1992	France	279 (85)	Pork tongue in jelly	4b
1993	France	38 (10)	Rillettes	4b
1996	Australia	5 (1)	Diced, cooked chicken	ND
1998–1999	US	108 (14)	Hot dogs	4b
1999	US	11 (ND)	Paté	ND
1999–2000	France	10 (3)	Rillettes	4b
1999–2000	France	32 (10)	Pork tongue in aspic	4b
2000	US	30 (7)	RTE deli turkey meat	^½^a
2000	New Zealand	30 (ND)	RTE deli meats	^½^a
2001	US	16 (ND)	Deli meats	^½^a
2002	US	54 (8)	RTE deli turkey meat	4b
2006–2007	Germany	16 (ND)	RTE scalded sausage	4b
2011	Switzerland	6 (ND)	Cooked ham	^½^a
2013–2014	Denmark	41 (7)	Meat products	ND
2017–2019	South Africa	1036 (216)	Polony	4b (ST6)

*Source:* Adopted from Jadhav, S., 2015, ‘Detection, subtyping and control of *Listeria monocytogenes* in food processing environments’, Doctoral dissertation, Melbourne, Swinburne University of Technology

RTE, ready-to-eat; ND: No record; US, United States; UK, United Kingdom.

## Pathogenicity of *Listeria monocytogenes*

**FIGURE 1:**
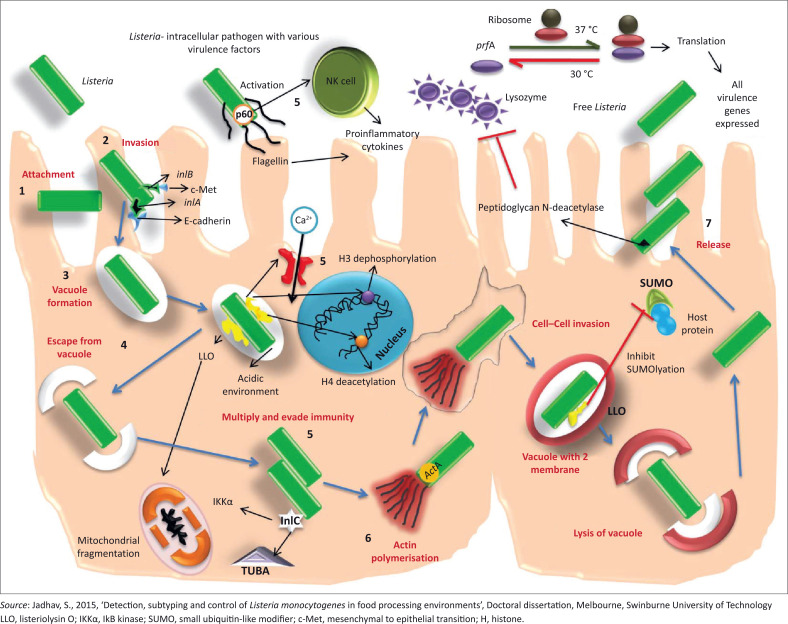
Pathogenesis and virulence genes involved in listeriosis infection in human cells.

The success of *L. monocytogenes* to induce infection is because of the ability to promote its own internalisation through host cells (Carvalho et al. 2014). This pathogen has the capacity to pass three important barriers in the human host, namely the intestinal epithelium, the blood–brain barrier and the placenta and subsequently disseminate to other organs (Chen et al. 2009). The infection process of host cell by *L. monocytogenes* involved several different stages: adhesion and invasion of host cells, internalisation by host cells, lysis of vacuole, intracellular multiplication and intercellular spread to the adjacent cell (Vazquez-Boland et al. 2001). Upon ingestion through contaminated food, *L. monocytogenes* survives exposure to high acidity, bile salts, non-specific inflammatory attacks and proteolytic enzymes from the host system (Jeyaletchumi et al. 2012). Having survived this stage, *L. monocytogenes* adheres to and enters both phagocytic and non-phagocytic cells of the host through the assistance of surface proteins called internalin (Carvalho et al. 2014). The phagocyte cells possess mechanisms that are used to destroy ingested bacteria; therefore, the ability of *L. monocytogenes* to survive within these cells contributes to its pathogenicity (Wilson et al. 2018). After adhering to the epithelial tissue of the gastrointestinal tract through assistance of internalin proteins, *L. monocytogenes* is internalised by the macrophages in a primary phagosomal vacuole. After it has been internalised, *L. monocytogenes* escapes from phagosomal vacuole through the assistance of cytolysin called listeriolysin O (LLO) and phosphatidylinositol-specific phospholipase (plcA). The pathogen then replicates in the cytoplasm because of sufficient nutrients from the host. An actin-based motility machinery of the host cell facilitates intracellular movement of the organism across the cytoplasm to neighbouring cells (Neves et al. 2013), thus spreading the infection without re-exposure to host extracellular immune surveillance (Vazquez-Boland et al. 2001). Bacterial surface protein called actin polymerisation protein (ActA) was identified as the molecular determinant for intracellular movement of the *L. monocytogenes* within the cytoplasm (Bonazzi, Lecuit & Cossart 2009). Upon being internalised by neighbouring cells, *L. monocytogenes* is confined in a double-membrane vacuole from which it escapes with the assistance of LLO and plcB to restart its life cycle as has been shown in Figure 1.

## Virulence factors of *Listeria monocytogenes*

*Listeria monocytogenes* consists of a large group of virulence factors that contribute to its pathogenicity and act in various steps of host infection cycle (Jeyaletchumi et al. 2012). The majority of virulence determinants of *L. monocytogenes* are clustered along the chromosome in genomic islands or *Listeria* pathogenicity island-1 (LIPI-1) (Denes et al. 2014). However, LIPI-3 and LIPI-4 have also been identified through whole genome sequencings (WGSs) to carry important virulence factors of *L. monocytogenes*. The LIPI-1 contains six virulence factors, which include internalin (Hain et al. 2012), phosphatidylinositol-specific phospholipase (Gilmour et al. 2010), actin polymerisation protein and metalloprotease (encoded by *mpl*) (Jeyaletchumi et al. 2012). The expression of these genes is controlled by the positive regulatory factor A (PrfA) (Rabinovich et al. 2012).

### Internalin

Adhesion and invasion of host cell by *L. monocytogenes* is the first step in intracellular life cycle. These steps are important for *L. monocytogenes* to cause disease in the host and are primarily mediated by two subfamilies of internalin proteins (Vazquez-Boland et al. 2001). The first subfamily is large surface proteins (70–80 kDa), such as *inlA* and *inlB* that attach to the bacterial cell through their C-terminal regions (Bonazzi et al. 2009). The second group is the smaller sized surface proteins (25 kDa – 30 kDa) such as inlC, inlD, inlE, inlF, inlG and inlH that lack the C-terminal cell-wall anchor region (Vazquez-Boland et al. 2001).

The adhesion of *L. monocytogenes* to the host and internalisation into a membrane bound vacuole is facilitated by *inlA* and *inlB* genes, which are encoded by *inlAB* operon (Hain et al. 2012; Hernandez-Milian & Payeras-Cifre 2014; Jeyaletchumi et al. 2012). The *inlA* is responsible for facilitating the binding between *L. monocytogenes* and the host adhesion protein E-cadherin for invasion of epithelial cells (Vazquez-Boland et al. 2001). An E-cadherin is a protein expressed on the surface of enterocytes (Lekkas 2016). This binding promotes local cytoskeletal rearrangements in the host cell to stimulate the uptake of *L. monocytogenes* by epithelial cells. The *inlB* binds the cellular receptor Met, a tyrosine kinase protein, which is also the endogenous ligand of the hepatocyte growth factor (Bonazzi et al. 2009). This binding allows *L. monocytogenes* to enter into a much wider range of host–cell types such as hepatocytes, fibroblasts and epithelioid cells (Sabet et al. 2005). The differences in expression of *inlA* and *inlB* are associated with poor invasion (Werbrouck et al. 2006) and mutations in *inlA*, which results in low invasion ability (Cases et al. 2016).

Other proteins of the internalin family include *inlC* and *inlJ* that are involved in post-intestinal dissemination of *L. monocytogenes* infection (Pournajaf et al. 2016). An *InlC* is produced after the *L. monocytogenes* has entered the host cell and functions to interact with IkB kinase (IKKα), which in turn prevents activation of nuclear factor-κB (NF-κB), a proinflammatory pathway (Liu et al. 2007) to dampen the host’s innate responses (Cases et al. 2016). An *InlJ* protein was discovered, as proteins that assist *L. monocytogenes* to cross the intestinal barrier of the host cell (Cases et al. 2016).

### Listeriolysin O and phospholipases

Once *L. monocytogenes* invades the host cell, it is trapped in the single-layer membrane vacuole. However, later in the infectious process, the bacteria are surrounded by a double-membrane vacuole (Yu et al. 2018). Escaping from both layers of vacuole membranes is important for an effective infection, and failure to escape the membrane results in an infection that is removed fast from the tissues (Pushkareva & Ermolaeva 2010). The haemolysin (*hly*), gene, is responsible for producing a pore-forming surface toxin called LLO, which is required for lysis of vacuole membranes and the release of *L. monocytogenes* into cytoplasm (Kyoui et al. 2014). The absence of LLO equals avirulent strains of *L. monocytogenes* as the bacterium will not reach the cytoplasm (Pushkareva & Ermolaeva 2010); thus, it can be said that LLO is secreted by all virulent strains of *L. monocytogenes*. The LLO is highly affected by the environmental pH with higher levels of expression observed under acidic pH levels (*pH* < 6) and lower activity levels observed at neutral pH (Pushkareva & Ermolaeva 2010).

Listeriolysin S is another virulence factor that was identified by Cotter et al. (2008). This virulence factor is located in LIPI3 and it is regarded as secondary haemolysin, which is specifically found only on lineage I strains of *L. monocytogenes*. This second haemolysin is only induced under oxidative stress conditions, contributes to virulence of the pathogen as assessed by murine (mice and rats) and human polymorphonuclear neutrophil-based studies and is similar to the peptide streptolysin S produced by *Streptococcus* (Cotter et al. 2008).

*Listeria monocytogenes* also secretes phosphatidylinositol phospholipase C that are involved in the lysis of vacuole membranes. Two phospholipases that assist in lysis of vacuole membranes are *plcA* (Gouin et al. 1994) and *plcB* (Vazquez-Boland et al. 2001). Studies have showed that *plcA* assists in the escape of *L. monocytogenes* from the primary vacuole, whereas *plcB* is active during cell-to-cell spread of the bacteria (Doyle et al. 2013). Maturation of *plcB* is dependent on a zinc metalloprotease, which is encoded by *mpl* gene. Metalloprotease also assists *hly, plcB* and *plcA* to disrupt the primary vacuoles after host cell invasion (O’Connor et al. 2010).

### Actin polymerising protein

*Listeria monocytogenes* reaches the cytoplasm after lyses of the vacuole and subsequently moves to infect other cells (Chatterjee et al. 2006). It achieves this by a surface protein called ActA that induces polymerisation of globular actin molecules to actin filaments. The filaments then facilitate movement of *L. monocytogenes* both inter- and intra-cellularly (Klumpp & Loessner 2013). Therefore, ActA is also required for *L. monocytogenes* pathogenicity (Vázquez-Boland et al. 2001), as it is critical in cytoplasmic movement of this pathogen. In a study performed by Doyle et al. (2013), using mice to compare the virulence of *L. monocytogenes* serotypes, the researchers observed lower virulence in serotypes 4a, 4c, 4d and 4e. The lower virulence in those serotypes was attributed to the production of lower levels of the ActA protein with actin tail formation. Serotypes 4a, 4c, 4d and 4e are considered avirulent strains because of lack of lower levels of ActA protein (Doyle et al. 2013).

### Invasion-associated protein (Protein p60)

The invasion-associated protein (iap) is an extracellular protein p60 that is encoded by *iap* gene (Camejo et al. 2011). Protein p60 is common amongst *Listeria* spp. and regarded as an essential murine hydrolase enzyme that facilitates septum separation during the final stage of cell division (Yu et al. 2018). It is also involved in adherence of *L. monocytogenes* to the host cell and plays an important role in virulence and pathogenicity of this bacterium (Quendera et al. 2016).

### Positive regulatory factor A

**FIGURE 2:**
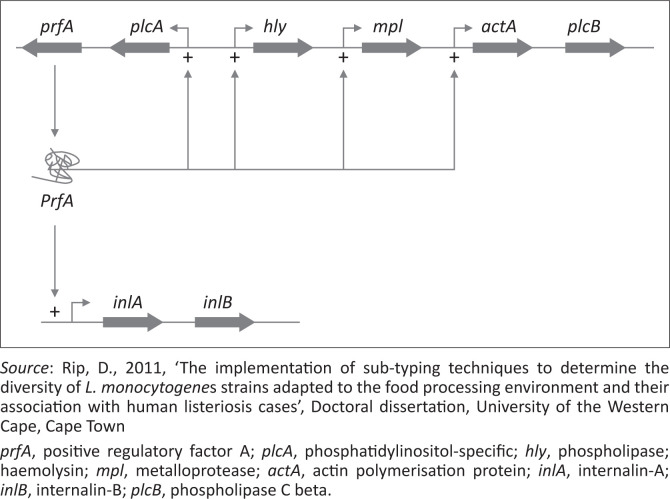
Organisation of the central virulence gene cluster of *Listeria monocytogenes*.

Expression of *prfA* is controlled in different ways either by *PrfA* itself or by an alternative sigma factor σ^B^ (Duroux et al. 2015). The temperature has an influence on the production of virulence factors because the secondary structure of untranslated *prfA*-mRNA is temperature dependent (Revazishvili et al. 2004). At 30 °C, the Shine-Dalgarno sequence is blocked and the ribosomes are not able to bind and translate the sequence. As a result of the positive feedback mechanism, only a small amount of *prfA* is therefore transcribed (Duroux et al. 2015). At 37 °C, the secondary structure has changed, which results in translation of *prfA*-mRNA followed by synthesis of *PrfA* and results in a higher amount of transcribed *prfA*. The *prfA* is the primary regulator of the expression of the virulence factors present in the virulence gene cluster (Figure 2), but other proteins act as virulence gene regulators too. The VirR is a response regulator critical for *L. monocytogenes* virulence (Ragon et al. 2008). The genes regulated by VirR encode ABC transporters, proteins involved in resistance to human defences in *Staphylococcus aureus* and cell wall modification proteins (Ragon et al. 2008). The alternative sigma factor σ^B^ is an overall regulator of the expression of several genes in response to several types of stresses, and it also regulates the expression of *PrfA* and thereby the expression of virulence factors. The *PrfA* is the primary regulator of the expression of the virulence factors present in the virulence gene cluster, but other proteins act as virulence gene regulators as well (Ryan et al. 2010).

## Epidemiology of human listeriosis

The cases of human listeriosis and the number of high profile outbreaks that resulted in many deaths had significantly increased in many countries (De Castro et al. 2012). The increase is mainly because of changing consumption behaviours, as many individuals consume RTE foods (Mateus et al. 2013). Furthermore, globalisation of food trade and demographic changes such as increase in susceptible populations because of ageing and existence of other immune-compromising infections have augmented the risk of listeriosis (Wang et al. 2012). The introduction of sequencing methodologies for detection and typing of listeriosis outbreaks has also led to increased number of cases being reported (Zuber et al. 2019).

Epidemiological surveillance studies showed that human listeriosis is mostly reported in high-income and industrialised countries because of proper surveillance system for food-borne diseases (Grace 2015). Table 2 shows the overall incidence of listeriosis per 100 000 people in different developed countries. Surveillance of human listeriosis performed by European Union (EU) between 2006 and 2012 on 18 Member States reported a notification rate of 0.41 cases per 100 000 population (Food and Authority 2013). The highest notification rates were observed in Finland, Spain and Denmark with a hospitalisation rate of 91.6% on an average (Food and Authority 2013). The surveillance also reported an increased incidence of listeriosis in Greece (0.3), Sweden (0.6), Norwary (1.0), France (0.6) and Scandinavia (0.2). This trend is accounted for by increased cases in the population older than 60 years and the higher consumption of smoked fish in these countries (Food and Authority 2013). Furthermore, the surveillance reported 198 deaths because of listeriosis from 18 Member States. Furthermore, the EU performed wide baseline survey in 26 Member States between 2010 and 2011 to determine the prevalence of *L. monocytogenes* in food products at retail outlets (Food and Authority 2013). A total of 13 088 food samples including smoked or gravad fish (3053), meat products (3530) and cheese types (3452) were tested for the presence of *L. monocytogenes*. The EU prevalence of *L. monocytogenes* varied amongst different food products with the highest recorded in fish (10.4%), followed by meat products (2.07%) and cheese (0.47%) (Food and Authority 2013).

**TABLE 2:** Incidences of listeriosis in different countries.

Countries/regions	Period	Incidence of listeriosis per 100 000 people	Reference
Canada	1990–1998	0.18–0.34	Todd and Notermans (2011)
England-Wales	1990–2000 2001–2009	0.21; 0.36	Jadhav (2015)
European Union	2000–2005	0.10–0.30	Jadhav (2015)
Belgium	2000–2005	0.43–0.86	Goulet et al. (2008)
Finland	2000–2005	0.35–0.79	Goulet et al. (2008)
Sweden	2000–2005	0.44–0.75	Goulet et al. (2008)
Germany	2001; 2005	0.26; 0.62	Jadhav (2015)
Switzerland	2001–2005	0.38–0.98	Goulet et al. (2008)
Netherlands	2002–2005	0.20–0.56	Doorduyn et al. (2006) Goulet et al. (2008)
US	2004–2009	0.25–0.32	Silk et al. (2013)
Denmark	2005–2008	0.52–0.85	Goulet et al. (2008)
New Zealand	2009–2010	0.50–0.60	Cruz et al. (2011)
Japan	2008–2011	0.14	Miya et al. (2015)

*Source:* Jooste, P., Jordan, K., Leong, D. & Alvarez-Ordóñez, A., 2016, Listeria monocytogenes in food: Control by monitoring the food processing environment. *African Journal of Microbiology Research* 10(1), 1–14

US, United States.

The notification rate of human listeriosis in the US was reported to be approximately 0.3 cases per 100 000 population (Todd & Notermans 2011). This rate was similar to that reported in Canada and New Zealand, which was lower compared with Europe (Food and Authority 2013). In the US, approximately 1 600 individuals get listeriosis annually with 21% case fatality rate (Todd & Notermans 2011). Almost all the fatalities were reported in high-risk groups, such as older adults, pregnant women and people who were immunocompromised. The Foodborne Disease Active Surveillance Network (FoodNet) had a surveillance system in 10 states of America for laboratory-confirmed cases of food-borne diseases (Rip 2011). This system revealed that there is a greater likelihood of being hospitalised from illness caused by *Listeria* than any other food-borne pathogen in the US (Rip 2011).

The OzFoodNet which is a working group established by the Australian Government and Department of Health and Ageing to survey listeriosis reported an annual (2001–2010) incidence rate of 0.3 cases per 100 000 population in Australia (Popovic, Heron & Covacin 2014). According to the Japan Nosocomial Infections Surveillance, approximately 135–201 cases of human listeriosis occurred annually between 2008 and 2011 (Miya et al. 2015), which suggests an incidence rate that is equivalent to 1.40 cases per 100 000 populations. Cases of human listeriosis have also been reported in other Asian countries such as Taiwan that reported 48 cases of listeriosis between 1996 and 2018 (Laksanalamai et al. 2014), whereas China reported 479 cases over a period of 46 years (1964–2010) (Jadhav 2015).

The World Health Organization (WHO) evaluated the median rate of listeriosis in different regions in 2010 and estimated that the incidence of listeriosis in Africa was 0.1 cases per 100 000 populations (Carp-Cǎrare, Vlad-Sabie & Floriştean 2013). However, the data in this evaluation were limited as many African countries neither report nor monitor the incidence of listeriosis because of lack or absence of targeted surveillance programmes and reporting systems (Grace 2015; Todd & Notermans 2011). Despite the poor surveillance programmes and lack of epidemiological data to establish a comprehensive incidence rate of human listeriosis in Africa, there are a number of studies that reported the occurrence of *L. monocytogenes* in foods from African countries (Table 3) (Bouayad & Hamdi 2012; Derra et al. 2013; El-Shenawy et al. 2011; Matle et al. 2019).

**TABLE 3:** Prevalence of *Listeria monocytogenes* in foods in Africa.

Authors	Country	Foodstuffs	Overall prevalence
Bouayad and Hamdi (2012)	Algeria	RTE dairy and meat foods (227 samples)	2.6% *L. monocytogenes*
Bouayad and Hamdi (2012)	Ethiopia	Retail meat and dairy products (240 samples)	4.1% *L. monocytogenes*
Ennaji et al. (2008)	Morocco	426 samples: (a) raw meat (*n* = 112), meat products (*n* = 240), poultry (*n* = 74)	2.4% *L. monocytogenes*
El-Shenawy et al. (2011)	Egypt	Street vended RTE food (576 samples)	14% *L. monocytogenes*
Morobe et al. (2006)	Botswana	Food samples from supermarkets and street vendors (1324 samples)	4.3% *L. monocytogenes*
Matle et al. (2019)	South Africa	Meat and meat products (2017 samples)	14.7% *L. monocytogenes*

*Source:* Jooste, P., Jordan, K., Leong, D. & Alvarez-Ordóñez, A., 2016, Listeria monocytogenes in food: Control by monitoring the food processing environment. *African Journal of Microbiology Research* 10(1), 1–14

RTE, ready-to-eat.

In SA, an average of 60 to 80 laboratory-confirmed listeriosis cases were reported annually before 2017 (Smith et al. 2019). This suggests an incidence rate of 0.1 cases per 100 000 populations, which is in agreement with that reported by WHO. However, in 2017, an increase in laboratory-confirmed cases of listeriosis was reported by National Institute for Communicable Diseases (NICD), which was linked to an outbreak. In 2018, the NICD had identified a total of 1024 laboratory-confirmed human cases across all nine provinces of SA that resulted in 216 deaths. The majority of cases were reported in the Gauteng province (59%) followed by the Western Cape (13%) and KwaZulu-Natal (7%) provinces. These cases were mostly reported in vulnerable groups, which includes infants (≤ 28 days), HIV-positive individuals, the elderly (> 65 years) and pregnant mothers (Smith et al. 2019).

The Agricultural Research Council of SA performed a comprehensive national baseline survey across nine provinces of SA including major ports of entry between 2014 and 2016 to determine the occurrence of *L. monocytogenes* in meat and meat products in abattoirs, meat-processing plants and retail outlets. Meat samples (*n* = 2017) consisting of raw meat, processed meat and RTE meat were analysed for *L. monocytogenes*. The occurrence of *L. monocytogenes* in meat destined for the South African market varied between imported (12.4%) and domestic (15.0%) meat samples, with the highest proportion reported in processed meat (19.5%), followed by RTE (13.5%) meat products and raw (10.1%) meat (Matle et al. 2019).

## Legislations relating to *Listeria monocytogenes* in food

Food-borne pathogens including *L. monocytogenes* remain a serious threat to public health and a significant impediment to socio-economic development worldwide (Grace 2015). Many countries responded to the threat posed by food-borne pathogens through implementation of strict regulations for microbiological standards or criteria in relation to contamination of food products (Strydom 2015). Microbiological criteria for food safety refer to the guidelines that are used to determine if a food product(s) is/are acceptable for human consumption based on the bacterial load of specific micro-organism on the food. The microbiological standards vary amongst different countries; however, they are guided by international standards such as International Commission on Microbiological Specifications for Food (ICMSF) and Codex Alimentarius Commission (CAC) (Rip 2011).

The ICMSF is a voluntary advisory organisation, which sets standards and methods regarding the presence of micro-organisms in food. The ICMSF states that food sample testing can be used for evaluation of safety and quality of food and to assess, validate the efficacy of microbial control measure such as Hazard Analysis and Critical Control Point (Rip 2011). The ICMSF recommends maximum of 100 cfu/g of *L. monocytogenes* in food at the time of consumption for non-risk consumers.

The CAC is a set of food standards, guidelines and codes of practice produced with the aim of protecting consumer health and facilitating international trade (Rip 2011). Compliance with the CAC standards is voluntary, but many governments and non-government institutions use the CAC guidelines as the basis for legislation and microbiological standards. The CAC standard on *L. monocytogenes* applies to only RTE foods, which divides them into three categories based on the ability of food product to support the growth of this pathogen (Food and Authority 2013). Microbiological criterion for *L. monocytogenes* in RTE foods belonging to the first category does not need to be determined. Therefore, microbiological criteria according to CAC have been set for RTE food products belonging to the second and third categories. The second group represents RTE foods that do not support the growth of *L. monocytogenes*, whereas the third group applies to the products that can support the growth of this pathogen. The acceptable level of 100 cfu/g has been set for food in the second category, whereas zero tolerance policy (absence of *L. monocytogenes* in 25 g of food) for products in the third category (Obaidat, Salman & Lafi 2015).

The European microbiological standard for *L. monocytogenes* in RTE food products is in accordance with CAC recommendations (Food and Authority 2013). The standard requires the absence of *L. monocytogenes* in RTE food products that are intended for infant consumption or as a medical food and in the food that can support the growth of this pathogen. However, in RTE food products that cannot support the growth of *L. monocytogenes*, the level < 100 cfu/g is required during the shelf life of those foods. Canada, Australia and New Zealand adopted microbiological standards similar to those of Europe as recommended by CAC (Jadhav 2015). The US has zero tolerance policy for *L. monocytogenes* in RTE food products and food-processing facilities and failure to comply is considered a serious offence (Piet et al. 2016).

In SA, meat safety is an important part of public health linking agriculture to health. The responsibility of ensuring meat safety is shared by two main national departments, namely the Department of Agriculture, Forestry & Fisheries (DAFF) and the Department of Health (DoH). The DAFF exercises authority over farms, feedlots and abattoirs and is mandated to administer the *Animal Diseases Act, Act 35 of 1984 and the Meat Safety Act, Act 40 of 2000* (Magwedere, Songabe & Dziva 2015). As soon as the meat leaves the abattoir supervision of distribution, retail and marketing falls in the hands of the DoH, which is entrusted with the *Foodstuffs, Cosmetics and Disinfectants Act* (*FCDA*), *Act 54 of 1972* (*as amended by Act 39 of 2007*). Section 2 (b) (i) of the FCDA does not allow any individual at food premises registered under this act to handle meat from an animal slaughtered in contravention of the *Meat Safety Act*. The FCDA forbids selling, manufacturing or importing for sale, any foodstuff that is contaminated, tainted or decayed or is in terms of any regulation deemed to be, detrimental to human health. However, these regulations are salient on microbiological criterion for *L. monocytogenes* in meat (Magwedere et al. 2015). The only microbiological criterion available for testing of meat in SA is the South Africa National Standard (SANS): 885:2011, which is non-mandatory and allows a maximum of 100 cfu/g for *L. monocytogenes* in RTE processed meat products.

## Occurrence of *Listeria monocytogenes* in meat value chain

**FIGURE 3:**
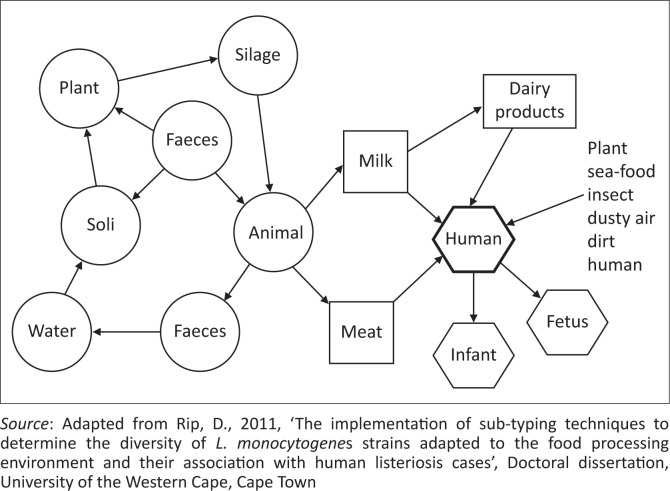
Implicated routes of transmission for *Listeria monocytogenes* infection to humans.

*Listeria monocytogenes* has been isolated from many different environments such as agricultural soil and vegetation (Susana et al. 2017), food-processing facilities (Martín et al. 2014) and retail outlets (Henri et al. 2016). Therefore, several sources have been identified as possible routes for contamination of food and subsequently transmission of *L. monocytogenes* to human beings. Contamination of meat and meat products with *L. monocytogenes* is a complex process that links primary food production (farm, feedlots) to retail outlets (Figure 3).

### *Listeria monocytogenes* in animal farm

In the farm or feedlots, *L. monocytogenes* is mostly found in soil as natural inhabitant but often at relatively low numbers (O’Connor et al. 2010). *Listeria monocytogenes* can survive in the soil especially in agricultural soil for months and even grow (Rip 2011). Sauders et al. (2012) reported a prevalence range from 8.7% to 51.4% for *L. monocytogenes* in agricultural soil, whereas in non-agricultural soils the prevalence ranged between 15.2% and 43.2%. This suggests that the soil serves as the principal reservoir of *L. Monocytogenes*, where meat-producing animals are exposed to this pathogen through interaction with the natural environment. Contaminated soil dust has also been found to contain this pathogen, suggesting that the animal can acquire *L. monocytogenes* through air and subsequently transmit it to human beings through the meat value chain (Korthals et al. 2008).

Several studies have associated animal listeriosis with consumption of contaminated silage by *L. Monocytogenes* (Korthals et al. 2008; Harakeh et al. 2009; Parihar 2004; Werbrouck et al. 2006). Silages are a traditional feed used to conserve animals during forage shortage because of seasonal changes in dry season (Zhu, Gooneratne & Hussain 2017). Therefore, a poorly prepared silage can support the growth of *L. monocytogenes* that can subsequently infect animals (Nightingale et al. 2004). Faeces shed by infected animals along with silage are vehicles for primary infection in ruminants and reintroducing of *L. monocytogenes* into the environment (Lekkas 2016). Asymptomatic shedding of *L. monocytogenes* by infected animals can also transmit this pathogen to human beings through food (Piet et al. 2016). Other sources of *L. monocytogenes* in a farm include poor animal husbandry, natural water and wastewater source. Linke et al. (2014) found that natural water and wastewater sources near farming communities harbour large quantities of *L. monocytogenes* and may serve as sources of animal contamination.

As *L. monocytogenes* is found naturally in the environment, pathogen control measures should therefore start from farm level up until the meat reaches the consumers’ table. Pre-harvest pathogen control includes all measures and management practices at farm level to reduce the probability of having pathogens in animals and final meat products (Nørrung & Buncic 2008). This should aim to minimise sources, access, levels and transfer of contaminants to the animal. Technologies used to reduce pathogen levels in animals include diet manipulation (supplements), effective biosecurity and optimum animal welfare (Sofos & Geornaras 2010). Proper animal management practices such as provision of clean water and feed and proper waste management to limit spreading of pathogens into the environment also help to reduce pathogen levels in animals (Buncic et al. 2014). However, it is difficult for farmers to control pathogens at this level because of lack of knowledge, resources and money in some cases.

### *Listeria monocytogenes* in food-processing facilities

*Listeria monocytogenes* has also been reported in different meat-processing facilities such as abattoirs, meat-processing plants and butcheries (Carpentier & Cerf 2011). Several studies suggest that animals presented for slaughter are an important source of initial contamination of meat-processing facilities with *L. monocytogenes* (Autio et al. 2000; Churchill et al. 2006; Lekkas 2016; Nigtingale et al. 2004). Autio et al. (2000) characterised different *L. monocytogenes* strains isolated from environmental samples in a pig farm and carcasses at abattoirs using pulsed-field gel electrophoresis (PFGE). The same pulsotypes were detected in a pig farm and carcasses, indicating that *L. monocytogenes* from farm might have contaminated the carcasses during production and processing cycle. Furthermore, Nel, Van Vuuren and Swan (2004) indicated that animals presented for slaughter often harbour large quantities of micro-organisms including *L. monocytogenes* on the external surface and hooves, which can be introduced in processing facilities if proper hygiene measures are not followed. In addition to animals, contamination may also enter the meat-processing facility through raw material and personnel carrying *L. monocytogenes* (El-Shenawy et al. 2011; Pava-Ripoll et al. 2012; Thévenot, Dernburg & Vernozy-Rozand 2006). Floors, floor drains, racks and rollers are also reported to be sources of contamination in meat-processing facilities (Thévenot et al. 2006).

Once *L. monocytogenes* enters the processing facilities it is unlikely to be eradicated if proper monitoring programmes are not intensive as its survival is influenced by several complex factors (Carpentier & Cerf 2011). The contributing factors to survival of *L. monocytogenes* include the ability to proliferate under harsh stress conditions such as low temperature, pH and osmotic stress (Takahashi et al. 2014), resistance to sanitation agents and formation of biofilms (Carpentier & Cerf 2011). Meat-processing facilities use cold storage (4 °C) to reduce the proliferation of bacteria on meat. Although this process is effective against many bacteria, it supports the growth of *L. monocytogenes* as this bacterium has the ability to survive at low temperature. The presence of cold shock proteins and sigma factor σ^B^, encoded by *sigB*, in *Listeria* spp., help them to survive in low temperature and osmotic pressure (Leong et al. 2014).

The occurrence of *L. monocytogenes* in meat-processing environment may also increase or decrease because of sanitation (Carpentier & Cerf 2011). Sanitation is a process that consists of cleaning protocols, followed by disinfection. Proper cleaning and sanitation procedures decrease the occurrence of *L. monocytogenes* in a meat-processing facility. However, this pathogen is often found even after cleaning. It shows the persistence of some strains and, on many occasions, the insufficiency of the cleaning (Cruz & Fletcher 2011). During the cleaning process, detergents are used to remove microbial agent on food-processing areas. However, this process is hampered by the presence of harbourage sites within the facility and concentration of sanitation (Lourenço, Neves & Brito 2009). Harbourage sites are areas where the sanitation agents do not reach correctly, and as a result of this it allows the proliferation of *L. monocytogenes.* Several studies showed that *L. monocytogenes* has the ability to resist commonly used sanitation agents in food-processing facilities (Bremer, Monk & Butler 2002; Fouladynezhad et al. 2013; Pan et al. 2006). These studies also reported that the resistance of *L. monocytogenes* to sanitising agents in food-processing facilities is because of the formation of biofilms and they subsequently persist in facility for several years. In fact, these studies showed that *L. monocytogenes* could form biofilms on surfaces such as polyethylene, polyvinyl chloride, glass and stainless steel in food-processing facilities. The formation of biofilms are influenced by characteristics of strains, physical and chemical properties of the substrate for attachment, growth phase of the bacteria, temperature, growth media and the presence of other microorganisms (Carpentier & Cerf 2011).

### *Listeria monocytogenes* in retail level

The detection of *L. monocytogenes* in meat and meat products at retail level does not mean that contamination occurred in the retail environment (Sauders et al. 2016). However, cross-contamination from surfaces, equipment and workers and persistence of strains have been identified as the main source of *L. monocytogenes* in retail products. Gombas et al. (2003) reported that meat products handled, sliced and packaged at retail outlets have high levels of *L. monocytogenes* than in products pre-packed at the abattoir. However, this depends on the level of hygienic practice followed by food handlers. Personal hygiene of meat handlers and proper sanitisation of contact surfaces and utensils are important to prevent cross-contamination or recontamination in retail outlets (Bogere & Baluka 2014). Storage temperatures should also be controlled to inhibit multiplication, surviving and growth of existing pathogens.

### *Listeria monocytogenes* in meat and meat products

Several studies have reported the existence of *L. monocytogenes* in meat and meat products originating from different animal species including game (Lambertz et al. 2012; Kramarenko et al. 2013; WHO & World Organisation for Animal Health 2014). In a study performed by Vitas and Garcia-Jalon (2004) in Spain that examined 396 meat product samples collected from 55 small meat-processing units identified *L. monocytogenes* in 34.9% of minced pork and beef meat products and in 36.1% of poultry meat. In Sweden, a survey of 507 heat-treated RTE meat product samples from 110 municipalities were analysed for *L. monocytogenes*. This pathogen was detected in 61% of heat-treated ham meat products, followed by 12% of turkey, 9% of roast beef and 7% of sausage (Lambertz et al. 2012). In Estonia, a survey baseline conducted over 10 years (2008–2010) indicated that *L. monocytogenes* was prevalent in 18.7% of raw meat and raw meat products, and 2% in RTE meat products collected from various food-processing facilities (Kramarenko et al. 2013). In Ireland, RTE meat samples analysed between 2013 and 2014 identified *L. monocytogenes* in 4.2% of meat products collected from poultry (Leong et al. 2014). Ismaiel, Ali and Enan (2014) reported *L. monocytogenes* in beef carcasses, raw lean beef, frozen chicken meat and Camel meat. Dhanashree et al. (2003) and Okutani et al. (2004) isolated *L. monocytogenes* in various meat products in India and Japan, respectively. Matle et al. (2019) reported *L. monocytogenes* in various meat and meat products including raw intact meat (10.1%), raw processed meat (19.5%) and RTE meat products (13.5%) collected from cattle, pork, sheep, game and poultry in SA.

### Diagnosis of *Listeria monocytogenes*

Identification of *L. monocytogenes* is extremely important for prevention and disease control. The method used for detection and isolation of *L. monocytogenes* has evolved over the years from cold enrichment technique to conventional and molecular methods (WHO & World Organisation for Animal Health 2014).

#### Conventional methods

Several conventional methods have been developed for isolation and identification *of L. monocytogenes* in food samples. The conventional method of isolation of *L. monocytogenes* includes antibody-based tests, enzyme-linked immunosorbent assay, culture-based methods and immune-capture techniques (Välimaa, Tilsala-Timisjärvi & Virtanen 2015). Out of these methods, culture-based tests are usually preferred for many reasons such as being sensitive, cheap and they remain the ‘gold standards’ compared with other methods that are validated (Barajas et al. 2019). In addition, pure colonies of the targeted organisms obtained by culture-based assays are useful for epidemiological surveillance and outbreak management purposes (WHO & World Organisation for Animal Health 2014). The drawbacks of culture-based methods include low resolution regarding distinguishing bacterial strains. Furthermore, these methods are laborious (Leong et al. 2014) and phenotypic changes because of environmental selection, contaminating bacteria and atypical reactions by atypical strains can provide false-negative results (Välimaa et al. 2015).

**FIGURE 4:**
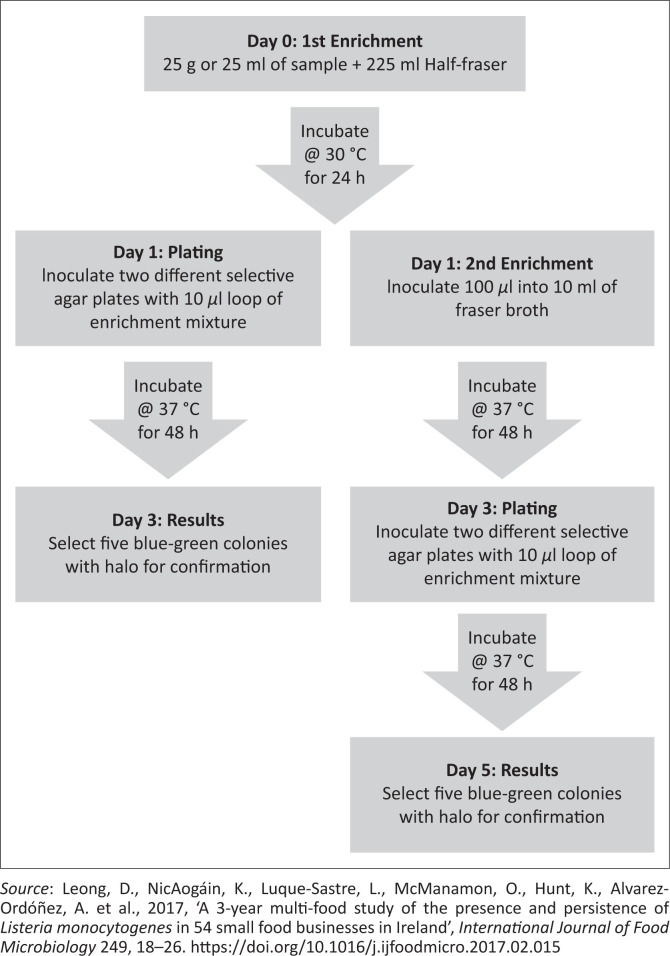
The illustration of International Standard method.

**FIGURE 5:**
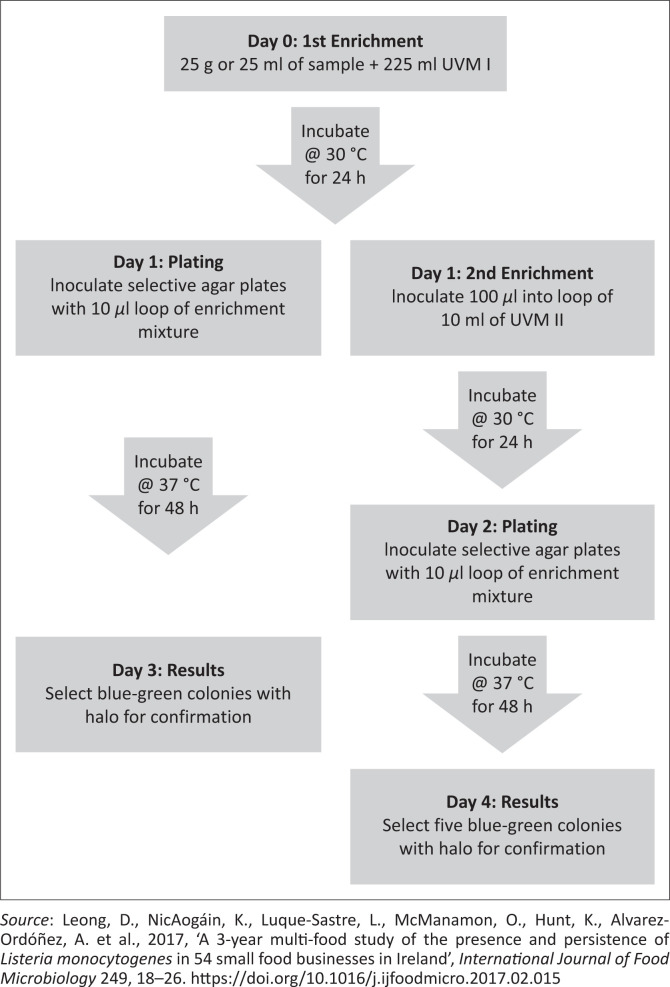
The illustration of United States Department of Agriculture method.

**FIGURE 6:**
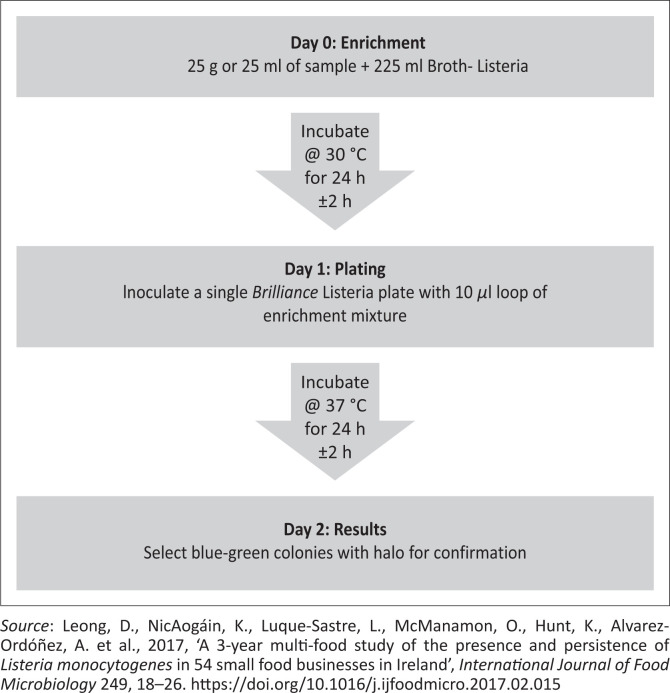
The illustration of ONE-Broth method.

The isolation and identification of *L. monocytogenes* using culture-based methods involve the use of selective agents and enrichment procedure. The purpose of the selective agents is to inhibit other competing microflora whilst the enrichment procedure allows the increase of *L. monocytogenes* to detectable levels and the recovery of injured or stressed cells (Chen et al. 2017). There are three commonly used culture-based methods (Figures 4–6) for isolation of *L. monocytogenes* in foods because of international regulations and requirements. These methods include the International Standard (ISO), the United States Department of Agriculture (USDA) and One-Broth *Listeria* method (Gasanov, Hughes & Hansbro 2005; Gómez et al. 2013; Zhang et al. 2004). Although these methods are internationally recommended and accepted for testing of a wide variety of food matrices, they must be used in accordance with their scope.

The ISO 11290 standard is recommended for isolation of *L. monocytogenes* in a large variety of food and feed products as well as environmental samples (Liu et al. 2007). The USDA method is recommended for the detection of *Listeria* spp. in meat and poultry food products and environmental swabs (Jeyaletchumi et al. 2012). One Broth *Listeria* method that has been approved by the Association Française de Normalisation (AFNOR) is used for isolation of *Listeria* spp. from dairy food products, meat, seafood and vegetables (Gasanov et al. 2005).

All above-mentioned methods involve a series of primary and secondary enrichments of the samples in selective broth media. The ISO 11290 method utilises Half Fraser Broth and Fraser Broth for primary and secondary enrichment, respectively. The USDA method uses a two-step enrichment in University of Vermont media, whereas the One Broth *Listeria* method uses a one-step enrichment in *Listeria* broth, which takes 2 days to produce results as opposed to the 5 and 4 days needed by ISO and USDA methods, respectively (Leong et al. 2017). These enrichment media contain different selective agents including cycloheximide, colistin, cefotetan, fosfomycin, lithium chloride, nalidixic acid, acriflavine, phenylethanol, ceftazidime, polymyxin B and moxalactam (Jadhav 2015). These antibiotics inhibit mostly the growth of Gram-negative bacteria that are often present as competitors in food samples. The mechanism of inhibition varies amongst these antibiotics, which includes inhibition of protein synthesis (cycloheximide, fosfomycin and nalidixic acid), disruption of the outer cell membrane of bacteria (colistin) and beta-lactamase (cefotetan) amongst others.

Following primary and secondary enrichment, the broth is generally plated onto selective or differential media. The ISO 11290 recommends the use of Oxford and PALCAM agar for detection and isolation of *L. monocytogenes.* The USDA uses chromogenic media such as Agar *Listeria* Ottaviani and Agosti and RAPID-L. mono (Leong et al. 2017), whereas One Broth *Listeria* method requires the use of *Listeria* Brilliance green agar. These media are typically dependent on the β-glucosidase activity of *Listeria*, which cleaves the chromogenic substrate producing blue or green colonies. Lecithin present in the agar is hydrolysed by phospholipase enzyme synthesised only by *L. monocytogenes*, leading to the formation of opaque halos around their colonies (Jeyaletchumi et al. 2012). Presumptive listerial colonies on selective agar are confirmed by rapid tests and on biochemical properties such as Gram stain, catalase test, motility test, ability to produce haemolysis on blood agar plates, Christie–Atkins–Munch-Peterson test with *Rhodococcusequi* and *Staphylococcus aureus* and carbohydrate utilisation tests.

## Molecular methods of detection

### Polymerase chain reaction-based methods for detection of Listeria monocytogenes

#### • Conventional polymerase chain reaction

The polymerase chain reaction (PCR) method has been used extensively for the detection of *L. monocytogenes*. Conventional PCR targets the most common and specific genes of *L. monocytogenes* such as *hly, inlA, inlB, iap, plcA, plcB*, 16S and 23S rRNA genes and dth-18 delayed type hypersensitivity protein (Jadhav 2015). The conventional PCR techniques are used more frequently than cultural procedures as they are simple and can provide quick results (Jeyaletchumi et al. 2012). However, this PCR cannot distinguish between live or dead cells or viable but not culturable cells (Truter 2015), metabolically injured, stressed cells or reliably detect low levels of *L. monocytogenes* (Jadhav 2015). Therefore, a positive sample through conventional PCR does not necessarily mean that the organism is alive and in required concentrations, which makes that organism a public health risk (Quendera et al. 2016).

#### Multiplex polymerase chain reaction

Multiplex PCR is another PCR-based method, which allows the detection of multiple strains from the same species or multiple pathogens in a sample simultaneously (Chen et al. 2017). The detection specificity of this method depends on the specific binding of the primer pair to the target sequence of the micro-organism. Ryu et al. (2013) developed a multiplex PCR method that can distinguish between five different *Listeria* spp. including *L. monocytogenes* by targeting different genes for each species. Multiplex PCR can detect between 1 and 100 cfu/mL *Listeria* (Jadhav 2015); however, similar to conventional PCR it can overestimate the presence of the pathogen because it cannot distinguish between live and dead cells.

#### Real-time polymerase chain reaction

Real-time PCR differs from other PCR techniques because the amplicon is observed as it accumulates. The procedure monitors the accumulation of fluorescence levels, which in turn depend on the amount of the accumulated PCR product. The fluorescent molecule can be either a target-specific probe labelled with a fluorescent dye together with a quencher molecule or can be a non-specific DNA-binding dye. The method is highly sensitive, can detect and trace amounts of target DNA, can be automated and has the ability to quantify bacterial load without any post-PCR handling. However, its disadvantages are that primer dimers can show fluorescence, it is highly dependent on primer concentration and design and it requires stringent quality controls.

### Subtyping of *Listeria monocytogenes*

Subtyping is a process that is used to discriminate amongst different bacterial strains that belong to the same species (Jeyaletchumi et al. 2012). Subtyping procedures are very useful for source identification and tracking of individual strains of *L. monocytogenes* that are involved in listeriosis outbreaks and to determine the population genetics, taxonomy and epidemiology of this pathogen (Doumith et al. 2004). There are many subtyping methods, which are broadly grouped as phenotypic and genotypic; however, not all will be discussed in detail in the current study, as they are not within the scope of this review. Those methods are listed in Table 4.

**TABLE 4:** Advantages and disadvantages of subtyping techniques used for *Listeria monocytogenes* strains.

Subtyping	Basis of discrimination	Advantages	Disadvantages	Reference
Phage typing	A technique used to subtype *L. monocytogenes* based on susceptibility to lysis by certain types of bacteriophage	Bacterial specific Easy to perform	Less discrimination capacity Its requirement for standardised phage panels	Jadhav (2015)
Multilocus Enzyme Electrophoresis	Differential electrophoretic mobility of bacterial enzymes because of differences in their amino acid composition	All strains are typeable using this technique	Time consuming and laborious	Djordjevic, Wiedmann and Mclandsborough (2002)
Amplified fragment length polymorphism	Polymorphism in DNA band sizes	Discriminative, and reproducible	Incomplete digestion of chromosomal DNA can lead to false results	Jadhav (2015)
Randomly amplified polymorphic DNA	Polymorphism in PCR amplification of genomic DNA by random primers	Rapid, simple and inexpensive	Non-specific annealing of primers can lead to inter-laboratory variations Less discriminative and less reproducible	Jeyaletchumi et al. (2012)
Ribotyping	A technique used to fingerprint ribosomal ribonucleic acid coding sequences	Highly reproducible and discriminative Automated version available	Inter-laboratory comparisons difficult Inefficient differentiation between ½b and 4b serotypes in some studies	Orsi et al. (2008)
REP and ERIC PCR	PCR-based methods that utilise primers which bind short repetitive extragenic palindromic elements	Rapid, inexpensive and simple technique Less laborious Species-specific primers do not have to be designed	Amplification between two REP/ERIC elements may not be genuine with use of low annealing temperatures Inter-laboratory variation	Morobe et al. (2006)

*Source*: Adapted from Jadhav, S., 2015, ‘Detection, subtyping and control of *Listeria monocytogenes* in food processing environments’, Doctoral dissertation, Melbourne, Swinburne University of Technology

REP PCR, repetitive extragenic palindromic sequence polymerase chain reaction; ERIC PCR, enterobacterial repetitive intergenic consensus polymerase; DNA, deoxyribonucleic acid.

#### Phenotypic subtyping methods

**Serotyping:** Serotyping is the first method used to differentiate *L. monocytogenes* strains from each other based on antigen–antibodies reaction (Ntivuguruzwa 2016). *Listeria monocytogenes* serotyping is performed using the slide agglutination method that characterise *L. monocytogenes* into 13 serotypes (^½^a, ^½^b, ^½^c, 3a, 3b, 3c, 4a, 4ab, 4b, 4c, 4d, 4e and 7) using unique combinations from somatic (O) and flagellar (H) surface antigens (antisera) reaction (Doumith et al. 2004). The value of this method in epidemiological studies is very limited because of poor discriminative powers (Gasanov et al. 2005). Serotyping is also associated with many drawbacks including failure to provide consistent or reliable and repeatable results, and it measures the phenotypic characteristics of *L. monocytogenes*, which are often subjected to change and does not always accurately reflect the genotype of a micro-organism (Liu 2006). In addition, serotyping is time consuming, difficult and requires high-quality antisera (Shaker & Hassanien 2015). Antigen sharing between *L. monocytogenes* and *L. Seeligeri* may lead to detection of incorrect serotype (Liu 2006).

#### Genetic sub-typing methods

**Polymerase chain reaction serogroup multiplex polymerase chain reaction assays:** To overcome the limitations of slide agglutination and enzyme-linked immunosorbent assay (ELISA) serotyping, a multiplex PCR-based method was then introduced by Borucki and Call (2003) for serotyping *L. monocytogenes* into five PCR serogroups; IIa corresponded to serotypes ½a and 3a; IIc to ½c and 3c; IIb to ½b, 3b and 7; and IVb to 4b, 4d and 4e (Doumith et al. 2004). This method uses primer pairs that target five genes (*lmo0737, lmo1118, ORF2110* and *ORF2819* and *prs*) to characterise *L. monocytogenes* strains and assign those PCR serogroups (Doumith et al. 2004; Nho et al. 2015). However, this assay could not distinguish between ½a and 3a, ½c and 3c, ½b, 3b and 7, 4a and 4c and between 4b, 4d and 4e, but as 3a, 3c, 3b, 7, 4a, 4c, 4d and 4e are rarely involved in human listeriosis, this approach was considered to be suitable for rapid detection of serotypes. Although this method is quick, robust and easy to implement, it has limited discriminatory power, thus providing poor resolution for epidemiological typing (Nho et al. 2015).

**Pulsed-field gel electrophoresis:** The PFGE is considered as ‘gold standard’ subtyping method for source tracking and epidemiologic investigations of infection caused by *L. monocytogenes*. This is because of its high discrimination power, robustness and reproducibility (Martín et al. 2014). However, it is time consuming and labour intensive, usually taking 2–3 days to complete and requires equipment of relatively high cost (Li et al. 2017). In PFGE, the genomic DNA is digested (generally using *AscI* and *ApaI* enzymes) with infrequent cutting endonucleases, which results in the generation of fewer fragments with high molecular mass (Kalpana & Muriana 2002). These fragments can be separated on the basis of their size using PFGE (Ruppitsch et al. 2015). The PFGE usually separates DNA fragments of less than 50 kb, as DNA fragments above 50 kb produce large and diffused bands (Jersek et al. 1999). In addition, it uses electric fields of alternating direction, which cause the DNA fragments to continuously change direction. This results in the resolution of high molecular weight DNA into separate bands (Hopkins, Arnold & Threlfall 2007).

**Multiple-locus variable number of tandem repeat analysis:** The multiple-locus variable number of tandem repeat analysis (MLVA) works on the principle to detect variation in the number of tandem repeats (VNTRs) at a specific locus in the genome DNA. The VNTRs are short segments of DNA that have variable copy numbers that can be determined by performing PCR amplification. The size of the PCR product is then analysed on agarose gels and/or by DNA-sequencing systems. The PCR product sizes are then used to determine the number of repeats in each region (Volpe Sperry et al. 2008). Therefore, by combining the size differences from several repeat loci regions, a multidigit, strain-specific code (profile) can be acquired, and these profiles can therefore be used for cluster analysis (Møretrø, Langsrud and Heir 2013; Murphy et al. 2007). The MLVA gained acceptance as subtyping method of bacterial isolates because of its robustness, rapidness and high discriminatory power (Camargo et al. 2016). Many authors suggested that MLVA can be a useful tool for the characterisation of *L. monocytogenes* strains and that it represents an attractive first-line screening method to epidemiological investigations and listeriosis surveillance (Chenal-Francisque et al. 2015; Lindstedt et al. 2008; Murphy et al. 2007). However, the main drawback of MLVA is the lack of reproducibility and its results cannot be compared amongst different laboratories. In addition, VNTRs are unstable and can even undergo change during routine laboratory subculturing and therefore affect the reproducibility of the MLVA method.

**Multilocus sequence typing:** Different multilocus sequence typing (MLST) methods have been described for *L. monocytogenes* typing (Den Bakker et al. 2013; Roberts et al. 2018). The MLST depends on multiple gene fragments or genes to differentiate between subtypes (Jeyaletchumi et al. 2012). The method relies on amplification of seven loci from housekeeping genes that are analysed for nucleotide differences. This method has been shown to be more discriminatory when compared against the gold standard for typing of *L. monocytogenes* PFGE method (Leong et al. 2017). Furthermore, MLST is considered an expensive and time-consuming method because it requires numerous sequencing reactions per isolate and cannot be multiplexed and it does not have enough discriminatory power for 4b *L. monocytogenes* serotypes (Jadhav 2015), which are amongst the serotypes often implicated in outbreaks. Direct interrogation of single-nucleotide polymorphisms (SNPs) could offer a more efficient alternative for DNA sequence-based subtyping based on the fact that the majority of sites sequenced for MLST are invariant (Ducey et al. 2006).

**Whole-genome sequencing:** Despite the fact that the phenotypic and molecular methodologies mentioned above have numerous advantages, those techniques, however, give limited information about the pathogenic organism because of discriminatory capacity (Lekkas 2016). The WGS has the power to overcome this hurdle and group isolates into epidemiologically relevant groups. The WGS has been recommended as new gold standard for subtyping of *L. monocytogenes* strain associated with outbreak (Fox et al. 2016). This technique has greatly improved since its inception, with the reduction in process time and the introduction of high-throughput next-generation sequencing technology. Furthermore, the technology is becoming cheaper and more user-friendly. In addition to epidemiological data, WGS can provide rapid generation of whole-genome sequence data that can help to identify targets that could be used to develop assays (Lekkas 2016).

Although the use of WGS provides valuable information that was not available previously in such a short time period, there are still major barriers that need to be addressed before these techniques can be incorporated in food-borne pathogen detection (Lekkas 2016). The need for computer platforms that are operator friendly, powerful enough to handle the massive data bases that are created and are easily interpreted still exists. In addition, there is no consensus on how these data will be stored or used by regulatory authorities such as FDA and CDC during inspections or outbreak investigations. It is almost certain that within the volume of data collected there will be some sequence data that might be misconstrued as indicating a health hazard (Eruteya & Odunfa 2014). Furthermore, limitations of the WGS approach are the need for highly trained bioinformatics professionals that smaller companies will not be able to afford, lack of standardised and validated protocols like the ones already existing for PFGE, lack of reference databases and lastly a large investment of resources that also have their own limitations (Orsi et al. 2011). In addition, food-borne pathogens are usually found at very low numbers, which poses its own limitations for epidemiological studies (Lekkas 2016).

## Treatment

Treatment of human listeriosis can be a challenging task as *L. monocytogenes* may invade almost all cell types (Dhama et al. 2015). Furthermore, the treatment of human listeriosis is often ineffective because of long incubation period of *L. monocytogenes*, which makes the treatment period to vary according to the level of the infection. However, antibiotics have been used to treat human listeriosis successfully for a very long time (Al-Nabulsi et al. 2015). *Listeria monocytogenes* is generally susceptible to the majority of antibiotics, but cephalosporin, fosfomycin and fluoroquinolones are not active against this pathogen (Noll, Kleta & Al 2018). The intrinsic resistance of *L. monocytogenes* against these antibiotics is because of lack or low affinity of enzyme catalysing the final step of cell wall synthesis (Al-Nabulsi et al. 2015). The antibiotic of choice for treating human listeriosis is ampicillin or penicillin G in combination with an aminoglycoside such as gentamicin. Trimethoprim in combination with a sulfonamide, such as sulfamethoxazole-co-trimoxazole, is considered second choice of therapy (Kovacevic et al. 2013). Furthermore, tetracycline, erythromycin and vancomycin have been used to treat human listeriosis (Rip 2011). However, evolution of bacteria towards resistance has been considerably accelerated in *L. monocytogenes* (Moreno et al. 2014).

## Antimicrobial resistance in *Listeria monocytogenes*

The acceleration in antimicrobial resistance of *L. monocytogenes* is linked to selective pressure exerted by over-prescription of drugs in clinical settings and their heavy use as promoters for growth in farm animals and increased global trade and travel, which favour the spread of antimicrobial resistance between countries and continents (Moreno et al. 2014; Zhang et al. 2004). Resistant *L. monocytogenes* strains have been reported against first-line antibiotics. Gentamicin-resistant clinical strains of *L. monocytogenes* were reported (Walsh et al. 2001). *Listeria monocytogenes* strain resistant to ampicillin was identified in the US (Verraes et al. 2013). *Listeria monocytogenes* resistant to streptomycin, erythromycin, kanamycin, sulfonamide and rifampin were also reported in clinical isolates in different countries (Moreno et al. 2014; Zhang et al. 2004). Multiple drug resistance has also been observed in strains isolated from foods and environmental samples across the world (Morobe et al. 2009). A study conducted in Northern Ireland (Walsh et al. 2001) showed 0.6% of *L. monocytogenes* from retail foods. Furthermore, antimicrobial resistance of *L. monocytogenes* isolated from food and animal sources (*n* = 167) in the US was determined. The resistance to ciprofloxacin, tetracycline, sulfonamide and nalidixic acid were 1.8%, 9%, 73% and 100%, respectively (Zhang et al. 2004).

### Mechanisms of antibiotic resistance in *Listeria monocytogenes*

*Listeria monocytogenes* becomes resistant to antimicrobial agents through acquisition of three types of movable genetic elements, namely self-transferable plasmids, mobilisable plasmids and conjugative transposons (Moreno et al. 2014). Efflux pumps were reported to be associated with fluoroquinolone resistance in *Listeria* (Wilson et al. 2018). However, there is an increase in reports of *L. monocytogenes* spontaneously acquiring resistant genes through mutations (Moreno et al. 2014). Mutations that occur in the promoter or operator coding regions can lead to overexpression of the endogenous genes such as those that encode for antimicrobial inactivating enzymes like the β-lactamase *AmpC*gene (Siu et al. 2003). Point mutations that occur in genes encoding for antimicrobial target regions can result in a target site that is resistant to the antimicrobial activity. Such a mutation was seen in the *gyrase* gene, whose mutation led to the expression of a fluoroquinolone-resistant *gyrase* enzyme (Hopkins et al. 2007).

### Antibiotic resistance mediated by conjugation

Conjugation is the process of transfer of genetic material, which occurs between living bacterial cells that are in direct contact (Verraes et al. 2013). Conjugation is the major mechanism used by *L. monocytogenes* strains to acquire antimicrobial resistance. *Enterococci* and *Streptococci*, in particular, represent a reservoir of resistance genes for *L. monocytogenes*. The gastrointestinal tract of humans is considered the most probable site where the acquisition by *Listeria* spp. of conjugative plasmids and transposons from *Enterococcus–Streptococcus* takes place (Wilson et al. 2018).

Charpentier and Courvalin (1999) reported that a broad host-range of plasmid pIP510 and pAMß1 initially found in *Streptococcus agalactiae* and *Enterococcus faecalis*, respectively, encoding resistance to chloramphenicol, macrolides, lincosamides erythromycin and streptogramins can be transferred by conjugation to *L. monocytogenes*. The Tn916, a broad host-range conjugative transposon that is primarily found in *Enterococcus faecalis* can also be conjugated from *E. faecalis* to *L. innocua* (Walsh et al. 2001). Conjugative transfer of the Tn916-related transposon Tn1545, initially found in *Streptococcus pneumoniae* was obtained from *E. faecalis* to *L. monocytogenes in vitro* and *in vivo* (Mata, Baquero & Pe 2000). Conjugative plasmids and transposons originating from *Enterococcus–Streptococcus* responsible for the emergence of resistance to tetracycline and chloramphenicol in *L. monocytogenes* have been reported (Walsh et al. 2001).

### Active efflux of antibiotics

Efflux mechanisms in *L. monocytogenes* was first reported in 2000 (Mata et al. 2000). The sequence of MdrL (multidrug efflux transporter of *Listeria*) protein is highly homological to the sequence of protein YfmO, a putative chromosomal multidrug efflux transporter of *Bacillus subtilis*. An allele-substituted mutant of this gene in *L. monocytogenes* failed to pump out ethidium bromide and presented increased susceptibility to macrolides, cefotaxime and heavy metals. Efflux pump Lde (*Listeria* drug efflux) is associated with fluoroquinolone resistance in clinical isolates of *L. monocytogenes* in France (Verraes et al. 2013). The Lde protein showed 44% homology with PmrA (pneumoniae multidrug resistance) of *Streptococcus pneumoniae*, which belongs to the major facilitator superfamily of secondary multidrug transporters. The insertional inactivation of the gene Lde resulted in increased susceptibility of fluoroquinolones in *L. monocytogenes* (Verraes et al. 2013).

### Alternative methods to control *Listeria monocytogenes*

Different alternative methods and therapies have been explored to reduce the presence of *L. monocytogenes* in foods, as there are few therapeutic options because of rapid development of antimicrobial resistance in this pathogen. The use of bacteriophage as biocontrol for *L. monocytogenes* and bacteriocins and essential oils in food, food-processing plants and humans has been reported (Klumpp & Loessner 2013; Soni et al. 2014). The use of probiotics has also been reported to inhibit growth of *L. monocytogenes* as they enhanced host immunity (Dhama et al. 2015).

Bacteriophages are viruses that can kill bacteria and were found as candidates for biocontrol of *L. monocytogenes* in meat and meat products during processing and packaging (Strydom 2015). Bacteriophages showed a high degree of specificity to lysis of *L. monocytogenes* strains without detrimental effects on normal microflora of the ultimate consumer and other desired bacteria in the food (Dhama et al. 2015). Furthermore, bacteriophages can self-perpetuate and they are stable during long cold storage (Strydom 2015). This suggests bacteriophages will be active against *L. monocytogenes* post-processing. Based on these desirable attributes, several commercial bacteriophage-based products have been developed for biocontrol of *L. monocytogenes* in food (Leong et al. 2017), which includes Phage LM-103a, phage LMP-102a, (Strydom 2015), Ply511 phage, ListShield, ListexP-100 and Listex^TM^ (Dhama et al. 2015). These products have been reported to function effectively against *L. monocytogenes* in foods and food facilities in different countries. For example, ListexP-100 and ListShield are used to control *L. monocytogenes* in the Netherlands and the US, respectively (Dhama et al. 2015). However, other countries such as SA still do not permit the use of bacteriophage-based products in food products and food-processing plants (Strydom 2015).

Bacteriocins are ribosomally synthesised antimicrobial peptides that can disrupt the integrity of the target cell membrane through forming pore on the membrane. The bacteriocins such as nisin have been used in meat products to inhibit the growth of *L. monocytogenes* (Rahimi et al. 2012). Bacteriocins have the potential to inhibit a wide variety of unrelated species or only closely related species. Recently nisin has been reported against *L. monocytogenes* and limited data have been generated to complete the understanding of the potential use for other bacteriocins for biocontrol of food-borne pathogens (Leong et al. 2017). Furthermore, essential oils from plant extracts have potential antimicrobial properties, which are suggested to reduce the survival of *L. monocytogenes* in various products. These essential oils include thyme, rosemary and oregano (Hilliard et al. 2018) and those from *Cinnamomum cuspidatum* and *Cinnamomum crassinervium* (Dhama et al. 2015).

## Conclusion

*Listeria monocytogenes* is amongst major food-borne pathogen in the world that has commanded most research and surveillance attention from government agencies and food industry over the last few years. Furthermore, methods for isolation, detection, identification and subtyping for *L. monocytogenes* from food products have increased rapidly with WGS as the new gold standard for typing of this pathogen. Despite the extensive research and development on *L. monocytogenes,* outbreaks associated with this pathogen continue to be reported and are exacerbated by a high number of susceptible individuals in most countries. However, there are no data on prevalence of *L. monocytogenes* from most African countries that are considered to have a significant population that is immunocompromised because of HIV, TB, malaria and other infectious diseases associated with poverty. Therefore, targeted surveillance programmes are necessary in those countries not only to determine prevalence but also for the development of regulations and microbiological criterion. In addition, increases in antibiotic resistance amongst *L. monocytogenes* strains are in line with a worldwide pattern of an increasing prevalence of antibiotic resistance amongst food-borne pathogens. Alternative therapies such as bacteriophages, bacteriocins and essential oil have been explored and show promising results.
